# Characterization and Enhanced Antioxidant Activity of the Cysteinyl β-Cyclodextrin-Baicalein Inclusion Complex

**DOI:** 10.3390/molecules21060703

**Published:** 2016-05-27

**Authors:** Hwanhee Kim, Hu Yiluo, Seyeon Park, Jae Yung Lee, Eunae Cho, Seunho Jung

**Affiliations:** 1 Department of Bioscience and Biotechnology, Microbial Carbohydrate Resource Bank (MCRB), Konkuk University, 120 Neungdong-ro, Gwangjin-gu, Seoul 05029, Korea; hwanhee@konkuk.ac.kr (H.K.); lannyhu0806@hotmail.com (H.Y.); 2 Department of Applied Chemistry, Dongduk Women’s University, Seoul 136-714, Korea; sypark21@dongduk.ac.kr; 3 Department of Biological Science, Mokpo National University, Jeonnam 534-729, Korea; jaeyunglee@gmail.com; 4 Center for Biotechnology Research in UBITA (CBRU), Institute for Ubiquitous Information Technology and Applications (UBITA), Konkuk University, 120 Neungdong-ro, Gwangjin-gu, Seoul 05029, Korea

**Keywords:** cysteinyl β-cyclodextrin, baicalein, inclusion complex, antioxidant activity

## Abstract

Baicalein is a type of flavonoid isolated from the roots of a medicinal plant, *Scutellaria baicalensis*. Although it has attracted considerable attention due to its antiviral, anti-tumor, and anti-inflammatory activities, its limited aqueous solubility inhibits the clinical application of this flavonoid. The present study aimed to prepare and characterize a host-guest complex in an effort to improve the solubility and antioxidant activity of baicalein. The host molecule is a macrocyclic β-cyclodextrin (β-CD) functionalized with cysteine for a synergetic effect. The structure of the synthesized cysteinyl β-CD was analyzed using nuclear magnetic resonance (NMR) spectroscopy and mass spectrometry. The inclusion complex with baicalein was studied by UV-vis, NMR spectroscopy, scanning electron microscopy, and X-ray powder diffractometry. The formed cysteinyl β-CD/baicalein inclusion complex efficiently improved the solubility and antioxidant ability of baicalein. Therefore, we suggest that the present cysteinyl β-CD is a potential host molecule for inclusion complexation and for bioavailability augmentation.

## 1. Introduction

In an inclusion complex system, macrocyclic host molecules are of great importance, as the cyclized and constrained conformation can provide the advantages of molecular selectivity and recognition [1]. The compound β-cyclodextrin (β-CD) is α-1,4-linked macrocyclic oligosaccharide containing seven glucose units. It can encapsulate hydrophobic compounds in its internal cavity [2]. Further, host-guest complexation can change the physico-chemical and biological characteristics of guest compounds. Due to its complex-forming ability, various applications are found in the pharmaceutical, food, and cosmetic industries [3]. 

To expand the applicability of β-CD, chemical derivatives such as hydroxypropyl and methyl β-CDs have been synthesized, and the inclusion complexing ability has been improved [4,5]. In addition, lipid derivatives of β-CD have been investigated as promising drug-carrier systems based on their self-assembled architecture [6,7]. However, few studies of amino acid or peptide derivatives have been reported to date [8,9]. Among them, cysteine modification is expected to provide antioxidant effects through the inactivation of hydroxyl radicals or through the support of glutathione synthesis [10,11].

Baicalein (5,6,7-trihydroxyflavone) is an active phytochemical isolated from Baikal skullcap root extract. It is used against inflammatory diseases such as hepatitis, nephritis, bronchitis, asthma, and atopic dermatitis [12]. In addition, antiviral [13], antibacterial [14], and anti-cancer activities have been found [15]. The beneficial effects on human health are also closely related to the antioxidant properties of baicalein because it has good hydrogen and electron donors, and the radical intermediates are stabilized by possible resonance forms [16]. Although interest in baicalein with regard to its chemopreventive properties has arisen, its poor aqueous solubility (90 µg/mL, 25 °C) inhibits its bioavailability. Thus, insolubility as a practical issue remains to be solved before additional clinical applications. 

Herein, we aim to synthesize cysteine-functionalized β-CDs for inclusion complexation with baicalein. Inclusion complexes were analyzed with nuclear magnetic resonance (NMR) spectroscopy, scanning electron microscopy (SEM), and X-ray powder diffractometry (XRPD). The modified properties of the complexed baicalein were also evaluated in terms of their aqueous solubility and antioxidant activities. The desired synergetic effect by the baicalein guest compound and the cysteine-conjugated host molecule was observed.

## 2. Results and Discussion

### 2.1. Structural Analysis of Mono-6-Cysteinyl-β-CD (Cysteinyl β-CD)

**Figure 1:**
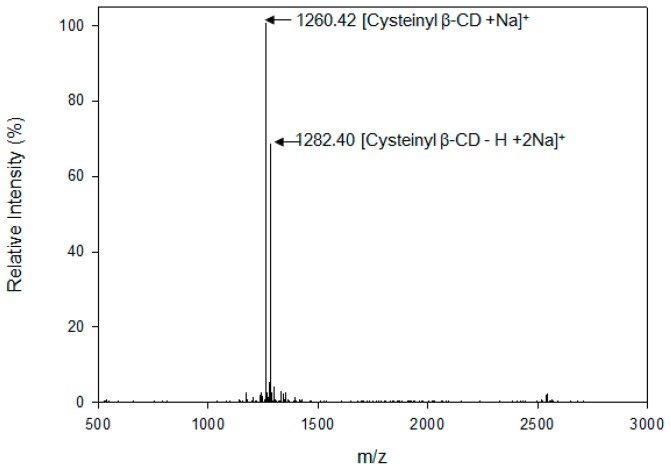
MALDI TOF mass spectrum of cysteinyl β-CD.

**Figure 2:**
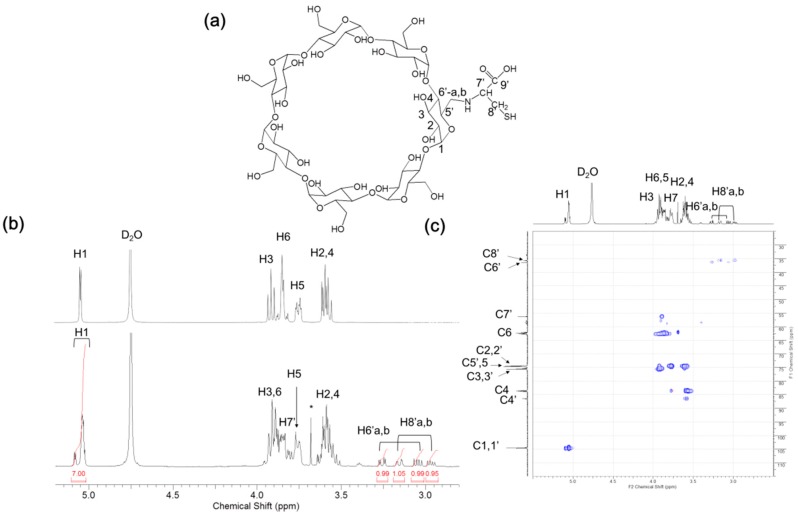
(**a**) Chemical structure of cysteinyl β-CD; (**b**) ^1^H-NMR spectra of β-CD (**top**) and cysteinyl β-CD (**bottom**); (**c**) HSQC spectrum of cysteinyl β-CD. Solvent: D_2_O.

**Scheme 1:**
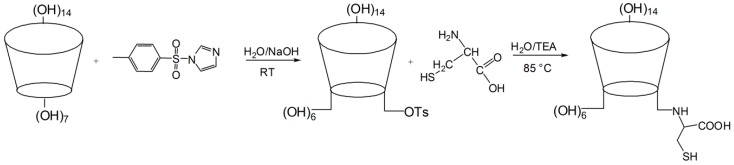
Schematic illustration of synthesis of cysteinyl β-CD.

Cysteinyl β-CD was prepared as described in the Materials and Methods (Scheme 1). After purification, the structure of the resultant product was analyzed using matrix-assisted laser desorption/ionization-time of flight (MALDI-TOF) mass spectrometry and NMR spectroscopy. Pseudo-molecular ion peaks ([mono-6-cysteinyl β-CD + Na]^+^ and [mono-6-cysteinyl β-CD − H + 2Na]^+^) were observed at *m*/*z* = 1260.42 and 1282.40 (Figure 1). The chemical structure is shown in Figure 2a. Due to cysteinyl modification, the anomeric protons of β-CD were also separated between δ 5.00 and δ 5.10. In the range of δ 2.90 to δ 3.30, upfield-shifted methylene protons on the C6 position of glucose were detected with the methylene signals of the side chain (Figure 2b). In its heteronuclear single quantum coherence (HSQC) spectrum (Figure 2c), compared to C6 carbons (δ 62.47) with a free OH group, the substituted C6′ carbons shifted significantly upfield to δ 36.48, becoming correlated with the methylene germinal H6′ signals. Chiral carbons of cysteine also appear at δ 56.38 with the cross-peak of the attached hydrogens (δ 3.73–3.95). Methylene carbon and hydrogen in the cysteine side chain were detected at δ 35.71/3.17 and δ 35.71/2.98, respectively. The results of these structural analyses indicate that cysteinyl β-CD was successfully synthesized.

### 2.2. Phase Solubility Diagram

**Figure 3:**
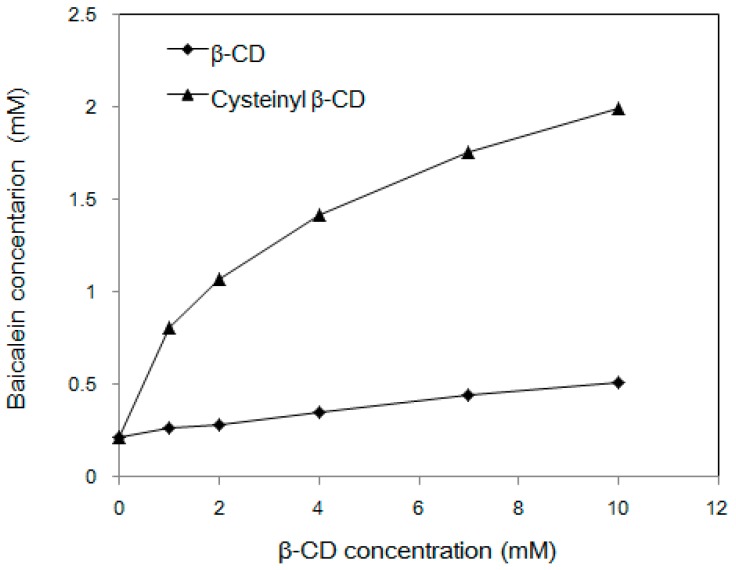
Phase solubility diagrams of baicalein with cysteinyl β-CD and β-CD in aqueous solution at 25 °C.

The substituted cysteine may provide additional effects related to electrostatic interaction and hydrogen bonding sites as well as easily oxidized thiol groups. Together with the attached β-CD cavity, cooperative recognition will lead to more sophisticated complexation for guest molecules. In relation to this, the inclusion capacity of cysteinyl β-CD for baicalein was investigated. The phase solubility diagram is shown in comparison with that of unmodified β-CD (Figure 3). According to Higuchi and Connors [17], an A_N_-type phase diagram for the cysteinyl β-CD/baicalein complex is obtained. Although cysteinyl β-CD-induced changes in the dielectric constant of the aqueous complexation solvent or self-association of the complexes will give rise to negative deviation from linearity, the curve indicates the formation of a soluble complex between cysteinyl β-CD and baicalein. The solubilizing efficiency (SE) is determined as the ratio between the solubility of the drug in the presence of a certain host concentration and the drug alone in water. Here, the solubilizing efficiency (SE 9.21) of cysteinyl β-CD was much better than that of the original β-CD (SE 2.35) in the presence of 10 mmol β-CDs. After attaching cysteine on β-CD, the intrinsic aqueous solubility of β-CD could be improved by about 100 fold (cysteinyl β-CD: >180 g/100 mL, 25 °C *vs.* β-CD: 1.82 g/100 mL, 25 °C). Furthermore, the complexation with baicalein may be more effective, although β-CD also has a capturable cavity.

### 2.3. NMR Studies

**Figure 4:**
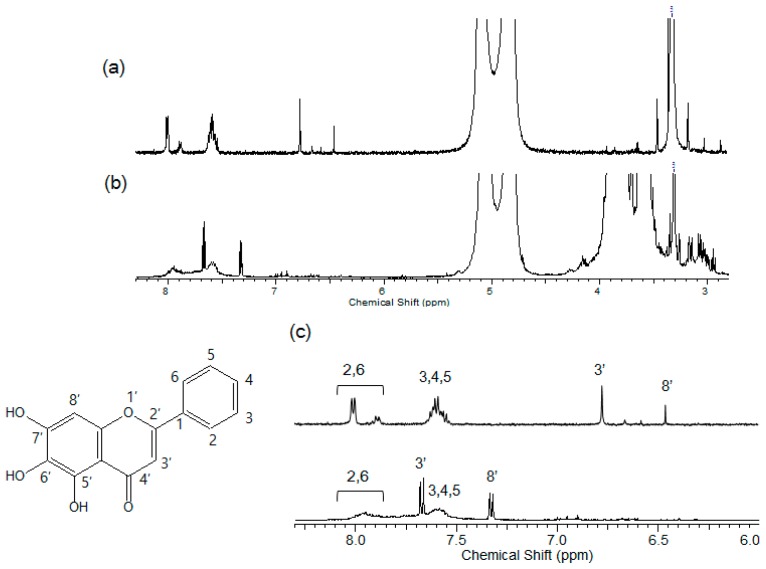
(**a**) ^1^H-NMR spectrum of baicalein (Solvent: D_2_O:MeOD, 70:30, *v*/*v*); (**b**) ^1^H-NMR spectrum of cysteinyl β-CD/baicalein complex (Solvent: D_2_O:MeOD, 70:30, *v*/*v*); (**c**) Enlarged spectrum of the aromatic region (top: baicalein, bottom: cysteinyl β-CD/baicalein complex). The inset shows the chemical structure of baicalein.

To examine non-covalent interactions at the molecular level, NMR spectroscopic analysis is performed. Figure 4 shows the ^1^H-NMR spectra, where the proton signals of baicalein are shifted in the upfield or downfield direction by the inclusion complex with cysteinyl β-CD. A downfield displacement indicates an environment of electronegative atoms [18], and an upfield shift displacement is attributed to the variation in the local polarity after insertion into a β-CD cavity [19]. The protons (2–6) in the benzyl ring are shifted toward the upfield direction, indicating the incorporation in the cavity. For the 3′ and 8′ protons in the chromene moiety, a pronounced downfield-shifted and split pattern is observed in the presence of cysteinyl β-CD. This result places stress on the chromene positioning toward the cysteine on the primary part of β-CD together with the benzyl insertion.

**Figure 5:**
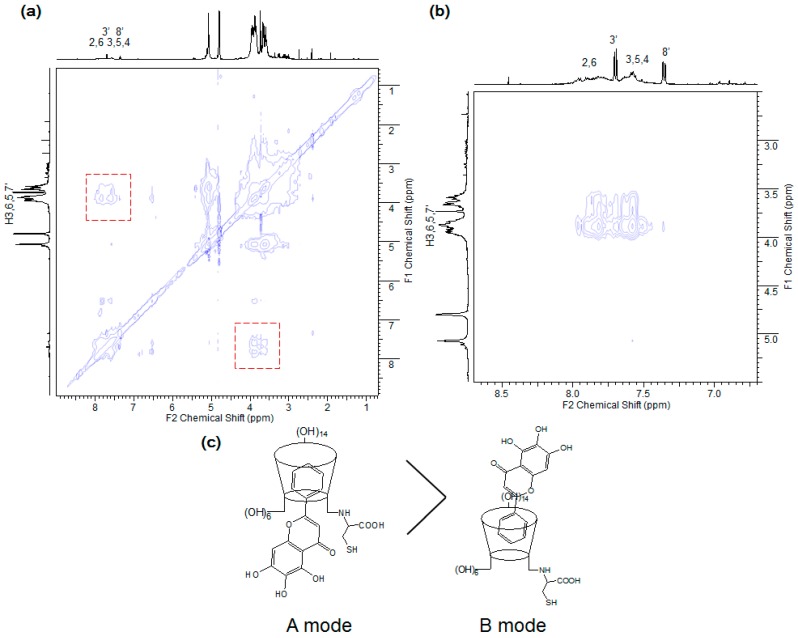
(**a**) NOESY spectrum of cysteinyl β-CD/baicalein complex (Solvent: D_2_O); (**b**) Enlarged spectrum of the area in the red rectangle; (**c**) The proposed model of the cysteinyl β-CD/baicalein complex.

In addition, two-dimensional nuclear Overhauser effect (NOE) signals were detected in a NOESY experiment. Given that two protons located in close proximity can induce an NOE cross-peak, this approach is useful for analyzing the intermolecular interaction of inclusion complexes [20]. Both the β-CD cavity (H3,5) and cysteine protons (H7′) share NOEs with benzyl-ring protons (2–6) and chromene protons (3′,8′) (Figure 5). Taken together, the results of these NMR spectroscopic analyses suggest that the plausible complex is the A mode rather than the B mode (Figure 5c). The benzyl moiety penetrates into the β-CD cavity from the narrow rim side, and the 5,6,7-trihydroxy-4*H*-chromene-4-one is located near the cysteine residue. The proposed structure would be effective for the cooperative complexation of baicalein and cysteinyl β-CD.

### 2.4. SEM Analysis

**Figure 6:**
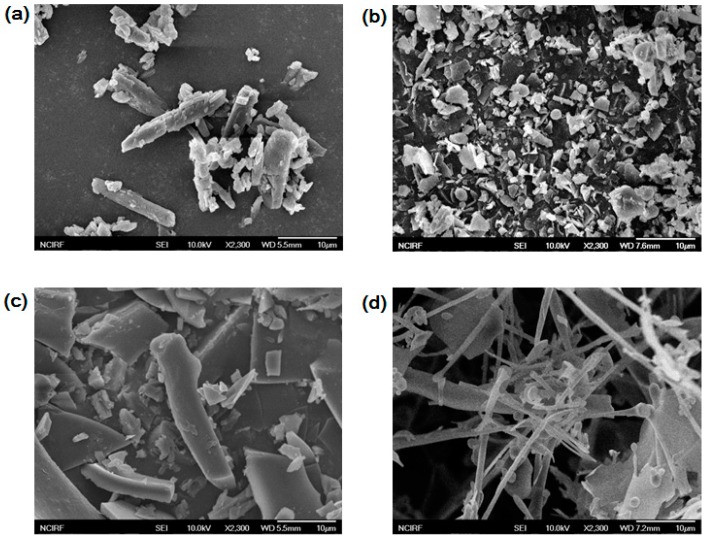
SEM photographs. (**a**) Baicalein; (**b**) Cysteinyl β-CD; (**c**) Physical mixture; (**d**) Cysteinyl β-CD/baicalein inclusion complex.

SEM analysis is frequently used as a tool to visualize the surface morphological changes in inclusion complexation studies [21,22]. Figure 6 shows the SEM images of baicalein, cysteinyl β-CD, the physical mixture, and the inclusion complex. Baicalein shows a rod shape of approximately 10 µm in length, whereas amorphous particles 2 µm in size are observed in cysteinyl β-CD. The physical mixtures showed a combined morphology of baicalein and cysteinyl β-CD. After complexation, the original morphologies of both compounds disappeared and fibrous structures were newly observed. Thus, the data obtained from the SEM analysis suggest that the inclusion complex formation process between baicalein and cysteinyl β-CD occurs in a solid state.

### 2.5. XRPD 

**Figure 7:**
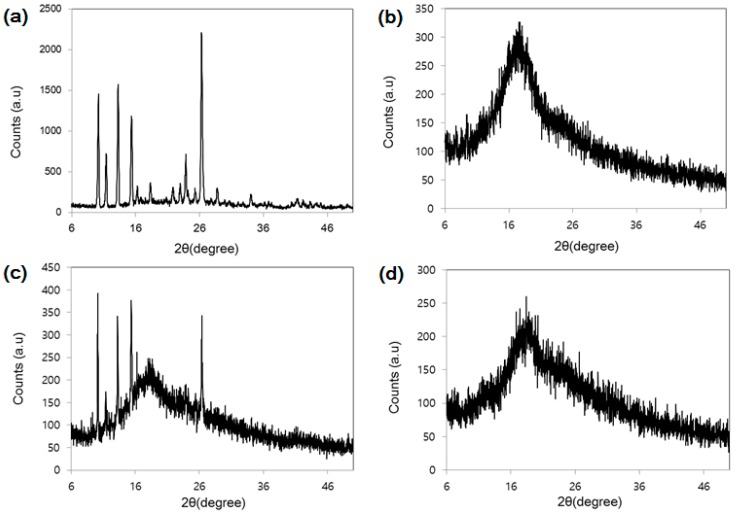
X-ray diffractograms. (**a**) Baicalein; (**b**) Cysteinyl β-CD; (**c**) Physical mixture; (**d**) Cysteinyl β-CD/baicalein inclusion complex.

Further evidence for the cysteinyl β-CD/baicalein inclusion complex is provided by XRPD experiments. XRPD is a suitable method for the evaluation of inclusion complexation in powder or microcrystalline states [23]. Figure 7 shows the disappearance of the sharp peak, indicating a lack of crystallinity with the formation of the inclusion complex. In the diffractogram of the physical mixture, a superimposed pattern of each compound is clearly displayed. Accordingly, the formation of the cysteinyl β-CD/baicalein inclusion complex is confirmed.

### 2.6. Antioxidant Effect 

**Figure 8:**
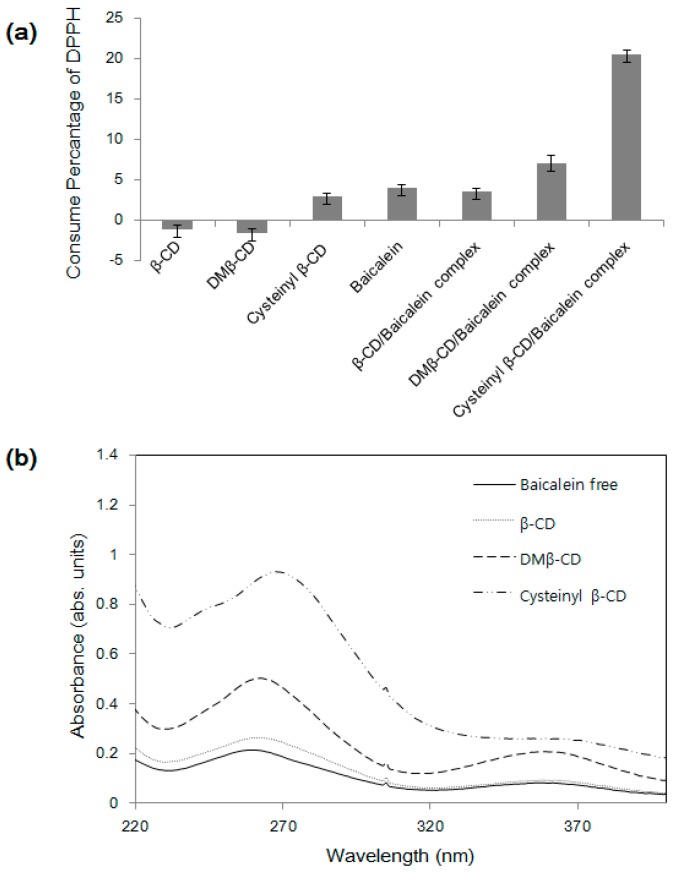
(**a**) Consumption of DPPH in the presence of baicalein, β-CDs and their complexes; (**b**) Absorption spectra of baicalein in aqueous solution with different β-CDs.

Baicalein has been reported to scavenge reactive oxygen species which cause damage to lipids, proteins, and DNA in cellular systems [24]. Based on the structure, one-step hydrogen atom transfer or an electron transfer followed by a proton transfer can be the antioxidant mechanism [16]. The antioxidant effects of samples were investigated based on a DPPH assay. Because DPPH is a stable free radical generating a violet color, a progressive discoloration represents antioxidant activity. The amount of DPPH free radical scavenging is estimated by observing the decrease in the absorbance at 518 nm [25]. The antioxidant activity of the cysteinyl β-CD/baicalein inclusion complex is clearly differentiated from that of free baicalein (Figure 8a). After efficient complexation, the limited antioxidant activity of baicalein was highly enhanced together with the aqueous solubility. The few negative values in the case of β-CD or 2,6-di-*O*-methyl-β-CD (DM β-CD) can be due to the enhanced solubility from the complex formation with the violet-colored DPPH in water [26]. Furthermore, cysteinyl β-CD shows little antioxidant activity. The synergetic antioxidant activity may be affected by the complexation mode (Figure 5c). The trihydroxy groups, the specific sites for the antioxidant effect, remain available to scavenge radicals, and the sulfhydryl group of cysteine can support this activity. Given that the β-CD cavity masks the irrelevant benzyl ring, the antioxidant activity of baicalein cannot be reduced, and, rather, a positive effect is considered by baicalein solubilization. Recently, the solubility of baicalein was investigated with the addition of α-, β-, γ-CD, hydroxypropyl-β-CD (HP β-CD), and DM β-CD, where DM β-CD showed the highest solubilizing effect [27]. Thus, the antioxidant activity of DM β-CD was also compared, and that of cysteinyl β-CD showed three-fold higher values compared to the antioxidant effect of DM β-CD under an identical treatment. The corresponding absorption spectra also support the antioxidant results (Figure 8b). Hence, we find that the synthesized cysteinyl β-CD is effectively utilized to improve the solubility and antioxidant activity of baicalein.

## 3. Materials and Methods

### 3.1. Chemicals

The β-CD (97%), 1-(*p*-toluenesulfonyl)imidazole (98%), and baicalein (>99%) were obtained from Tokyo Chemical Industry Co., Ltd. Triethylamine (TEA), DM β-CD, l-cysteine (97%), 2,2-diphenyl-1-picrylhydrazyl (DPPH), ethanol (>99.8%) were purchased from Sigma–Aldrich Chemicals Co. The used water was triply distilled. D_2_O (99.96% at D) and CD_3_OD (99.8% at D) were from Cambridge Isotope Laboratories, Inc. (Andover, MA, USA).

### 3.2. Synthesis of Cysteinyl β-CD

The cysteinyl β-CD was synthesized from mono-6-*O*-*p*-toluenesulfonyl-β-CD (tosyl β-CD), the most important intermediate for mono-functionalization on the primary side of β-CD [28]. The tosyl β-CD was synthesized as described in our previous report [29]. The β-CD (5.0 g, 4.4 mmol) was dissolved in water by heating it to 60 °C, and the cooled solution was mixed with 1-(p-toluenesulfonyl)imidazole (3.9 g, 17.7 mmol). After 6 h, a NaOH aqueous solution was added over a time of 20 min, and the unreacted precipitates were removed. Ammonium chloride (6.1 g, 115 mmol) was added to stop the reaction, and the mixture was concentrated to half of the original volume by blowing air. Tosyl β-CD began to precipitate, and the precipitates were washed and dried. Tosyl β-CD (921 mg, 0.7 mmol) and l-cysteine (251 mg, 2.1 mmol) were dissolved in a 40% (*v*/*v*) TEA aqueous solution (10 mL) at 85 °C while stirring for two days under N_2_. After 48 h, excess solvent was removed by evaporation under reduced pressure, and the concentrated sample was precipitated using ethanol (100 mL). The precipitate was collected, and the cysteinyl β-CD was purified using DEAE-Sephadex A-25 and a Bio-gel P-2 column. The lyophilized product was obtained at a 30% yield starting with β-CD.

*Mono-6-cysteinyl-β-CD*. ^1^H-NMR (500MHz, D_2_O): δ 5.10–5.00 (m, 7H, H1), 3.95–3.73 (m, H3,6,7′,5), 3.65–3.50 (m, H2,4), 3.29–3.23 (m, 1H, H6′a), 3.19–3.12 (m, 1H, H8′a), 3.08–3.01 (m, 1H, H6′b), 3.00–2.93 (m, 1H, H8′b); ^13^C-NMR (500 MHz, D_2_O): δ 175.26 (C9′), 104.64 (C1,1′), 86.49 (C4′), 83.54 (C4), 75.60 (C3), 75.29 (C3′), 74.63 (C5′), 74.58 (C5), 74.50 (C2), 74.44 (C2′), 62.47 (C6), 56.38 (C7′), 36.48 (C6′), 35.71 (C8′); MALDI-TOF MS: 1260.42 [mono-6-cysteinyl β-CD + Na]^+^.

### 3.3. MALDI-TOF Mass Spectrometry

MALDI-TOF MS (Voyager- DETM STR Bio-Spectrometry, Applied Biosystems, Framingham, MA, USA) was carried out to obtain the mass spectrum of cysteinyl β-CD in the positive ion mode. The 2,5-Dihydroxybenzoic acid (DHB) was used as the matrix.

### 3.4. NMR Spectroscopy

For the NMR spectroscopic analysis, a Bruker Avance 500 spectrometer (Bruker, Karlsruhe, Germany) was used to record the ^1^H-NMR, ^13^C-NMR, HSQC, and NOESY spectra. The NOESY spectra were recorded with 256/2048 complex data points using a pulse train to achieve a spin-lock field with a mixing time of 600 ms for the complex. 

### 3.5. Phase Solubility Studies

Baicalein was dissolved in methanol to obtain a 20 mM stock solution. In this case, 500 µL of the stock solution was added to an aqueous solution (500 µL) of β-CD and cysteinyl β-CD with various concentrations (0.0–10.0 mM) in capped vials. In each case, the mixture was magnetically stirred at 25 °C and shielded from light to prevent the degradation of the baicalein. After 72 h, methanol was evaporated, lyophilized, and dissolved in 500 µL water. After filtering (PTFE 0.2 µm filter, ADVANTEC), the solubilized baicalein was evaluated at 270 nm using a spectrophotometer (UV 2450, Shimadzu Corporation, Shimadzu, Japan). 

### 3.6. Aqueous Solubility of β-CD and Cysteinyl β-CD

The intrinsic solubility measurement was performed with some modification in the previous protocol [30]. The β-CD and cysteinyl β-CD were added to 250 µL of water, and the mixture was sonicated for 1 min and then stirred for 1 h at 25 °C. After lyophilizing the soluble fraction, the mass was measured as a function of the volume. Because of increasing viscosity, a more accurate estimation of solubility was difficult.

### 3.7. Preparation of the Cysteinyl β-CD/Baicalein Complex

Inclusion complexes of baicalein and cysteinyl β-CD were prepared using the suspension method [31]**.** Baicalein was dissolved in methanol and then added to an aqueous solution of an appropriate concentration of cysteinyl β-CD. The resulting mixture was stirred at 25 °C for 72 h. After equilibration, methanol was evaporated and lyophilized. The dissolved sample was lyophilized and the sample was then dissolved in 500 µL water, after which it was filtered and freeze-dried.

### 3.8. SEM

The surfaces morphology of the materials was examined using a field emission scanning electron microscope (JSM-6700F, JEOL, Ltd., Tokyo, Japan). The samples were precisely fixed on a brass stub using double-sided adhesive carbon tape, and were then made to be electrically conductive by sputter coating with a thin layer of gold in a vacuum. Images were taken at an accelerating voltage of 10 kV, and a magnitude level of 2300× was used.

### 3.9. XRPD

XPRD patterns for baicalein, cysteinyl β-CD, the physical mixtures and the inclusion complexes were traced employing high resolution X-ray diffractometer (Bruker D8 DISCOVER, Karlsruhe, Germany) at a scanning rate of 2°/min. The voltage/current used was 40 kV/40 mA and the angular range (2θ) covered was between 6° and 50°. 

### 3.10. Antioxidant Activity

The antioxidant activities of the samples are measured in terms of their radical scavenging ability (RSA), using the DPPH method [32]. A volume of 1 mL (ethanol:water, 50:50 *v*/*v*) of 50 µM DPPH was used. The reaction was started with an addition of 10 µL of each sample without and with a 2 mM β-CD concentration. The resulting mixtures were incubated in the dark at 25 °C. After the system reached equilibrium at the end of 30 min, the absorbance levels of the solutions were measured by UV-Vis spectroscopy at 518 nm. The results were expressed as the percentage of DPPH eradication calculated according to the following equation:

(1) $$\text{RSA}\;=\;(1\;-\;A_{S}/A_{B})\;\times \;100$$

where the absorbance of the sample is A_S_ and that of the blank sample is A_B_.

## 4. Conclusions 

In the present study, mono-6-cysteinyl-β-CD was successfully synthesized by reacting mono-6-*O*-*p*-toluenesulfonyl-β-CD with L-cysteine in water. The complexation characteristics of cysteinyl β-CD and baicalein were analyzed using UV-vis, NMR spectroscopy, SEM, and XRPD. The driving forces for the complexation are considered as non-covalent interactions such as hydrogen bonding, hydrophobic interaction, and van der Waals interactions between mono-6-cysteinyl-β-CD and baicalein. The results of NMR spectroscopic analyses show a complexation mode where the benzyl moiety penetrates into the β-CD cavity and the chromene part is located near the cysteine. This cooperative complexation can enhance the solubility and antioxidant activity of baicalein. Cysteinyl β-CD and this synergetic approach for inclusion complexation will be useful for the design and development of novel host molecules.
